# Author Correction: DNA methyltransferase DNMT3a contributes to neuropathic pain by repressing *Kcna2* in primary afferent neurons

**DOI:** 10.1038/s41467-020-18562-x

**Published:** 2020-09-14

**Authors:** Jian-Yuan Zhao, Lingli Liang, Xiyao Gu, Zhisong Li, Shaogen Wu, Linlin Sun, Fidelis E. Atianjoh, Jian Feng, Kai Mo, Shushan Jia, Brianna Marie Lutz, Alex Bekker, Eric J. Nestler, Yuan-Xiang Tao

**Affiliations:** 1 Department of Anesthesiology, New Jersey Medical School, Rutgers, The State University of New Jersey, Newark, New Jersey 07103 USA; 2 State Key Laboratory of Genetic Engineering, School of Life Sciences, Fudan University, Shanghai, 200438 China; 3 Department of Anesthesiology, The First Affiliated Hospital of Zhengzhou University, Zhengzhou, 450052 Henan China; 4 Department of Anatomy, College of Medicine, Howard University, Washington DC, 20059 USA; 5 Fishberg Department of Neuroscience and Friedman Brain Institute, Icahn School of Medicine at Mount Sinai, New York, New York 10029 USA; 6 Departments of Cell Biology & Molecular Medicine and Physiology, Pharmacology & Neuroscience, New Jersey Medical School, Rutgers, The State University of New Jersey, Newark, New Jersey 07103 USA

Correction to: *Nature Communications* 10.1038/ncomms14712, published online 8 March 2017.

The original version of this article contained an error in the author affiliations. Yuan-Xiang Tao was incorrectly associated with Department of Anesthesiology, The First Affiliated Hospital of Zhengzhou University, Zhengzhou 450052, Henan, China. This has not been corrected in both the PDF and HTML versions of the article.

