# Supplementary material for: DNA methyltransferase DNMT3a contributes to neuropathic pain by repressing Kcna2 in primary afferent neurons
Source: Nat Commun. 2017 Mar 8;8:14712. doi: 10.1038/ncomms14712 (PMC5344974; doi:10.1038/ncomms14712)
Supplement: Supplementary Information — Supplementary Figures and Supplementary Tables [file ncomms14712-s1.pdf]

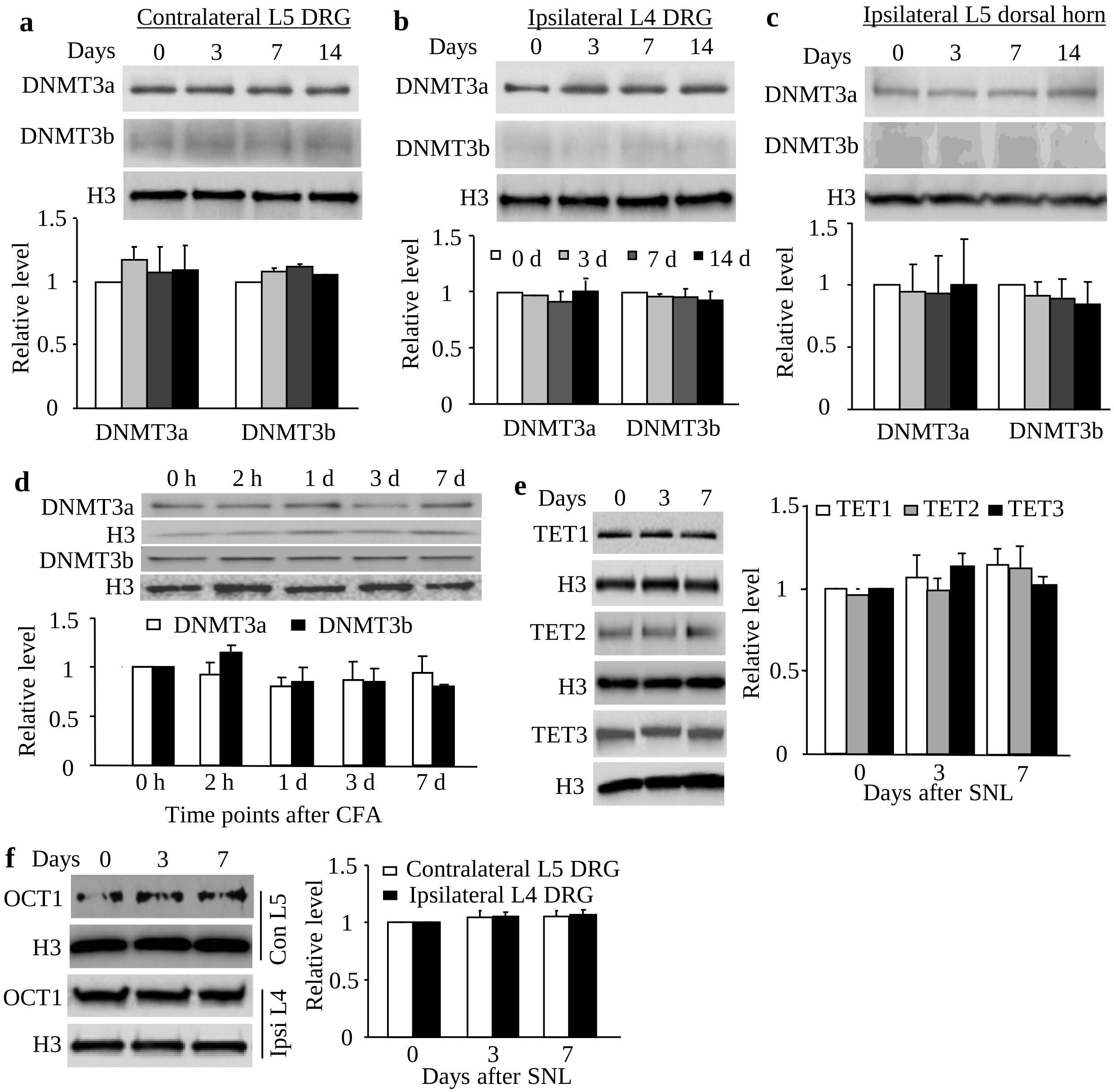
a
Contralateral L5 DRG
Days 0 3 7 14
DNMT3a
DNMT3b
H3
Relative level
1.5
1
0.5
0
DNMT3a
DNMT3b
b
Ipsilateral L4 DRG
Days 0 3 7 14
DNMT3a
DNMT3b
H3
0 d 3 d 7 d 14 d
Relative level
c
Ipsilateral L5 dorsal horn
Days 0 3 7 14
DNMT3a
DNMT3b
H3
Relative level
d
0 h 2 h 1 d 3 d 7 d
DNMT3a
H3
DNMT3b
H3
DNMT3a DNMT3b
Relative level
Time points after CFA
e
Days 0 3 7
TET1
H3
TET2
H3
TET3
H3
TET1 TET2 TET3
Relative level
Days after SNL
f
Days 0 3 7
OCT1
H3
OCT1
H3
Con L5
Ipsi L4
Contralateral L5 DRG
Ipsilateral L4 DRG
Relative level
Days after SNL

Supplementary Figure 1. (a) Levels of DNMT3a and DNMT3b in the contralateral L5 DRG on days 0, 3, 7, and 14 post-SNL in rats. n = 6 rats/time point. One-way ANOVA (relative level *vs.* time points) followed by *post hoc* Tukey test, F_{time} (3, 11) = 0.10 for DNMT3a and F_{time} (3, 11) = 0.27 for DNMT3b. (b) Levels of DNMT3a and DNMT3b in the ipsilateral L4 DRG on days 0, 3, 7 and 14 post-SNL in rats. n = 6 rats/time point. One-way ANOVA (relative level *vs.* time points) followed by *post hoc* Tukey test, F_{time} (3, 11) = 0.18 for DNMT3a and F_{time} (3, 11) = 0.17 for DNMT3b. (c) Levels of DNMT3a and DNMT3b in the ipsilateral L5 dorsal horn on days 0, 3, 7 and 14 post-SNL in rats. n = 6 rats/time point. One-way ANOVA (relative level *vs.* time points) followed by *post hoc* Tukey test, F_{time} (3, 15) = 0.87 for DNMT3a and F_{time} (3, 11) = 0.001 for DNMT3b. (d) Levels of DNMT3a and DNMT3b in the ipsilateral L4 and L5 DRG at 0 h, 2 h, 1 d, 3 d, and 7 d post-CFA in rats. n = 6 rats/time point. One-way ANOVA (relative level *vs.* time points) followed by *post hoc* Tukey test, F_{time} (4, 29) = 0.76 for DNMT3a and F_{time} (4, 14) = 1.49 for DNMT3b. (e) Levels of TET1, TET2, and TET3 in the ipsilateral L5 DRG on days 0, 3, and 7 post-SNL in rats. n = 12 rats/time point. One-way ANOVA (relative level *vs.* time points) followed by *post hoc* Tukey test, F_{time} (2, 8) = 0.70 for TET1, F_{time} (2, 8) = 0.76 for TET2, and F_{time} (2, 8) = 1.10 for TET3. (f) Level of OCT1 in the contralateral (Con) L5 DRG and ipsilateral (Ipsi) L4 DRG on days 0, 3, and 7 post-SNL in rats. n = 6 rats/time point. One-way ANOVA (relative level *vs.* time points) followed by *post hoc* Tukey test, F_{time} (2, 8) = 1.34 for Con L5 DRG and F_{time} (2, 8) = 0.66 for Ipsi L4 DRG. Full-length blots are presented in Supplementary Figure 6.

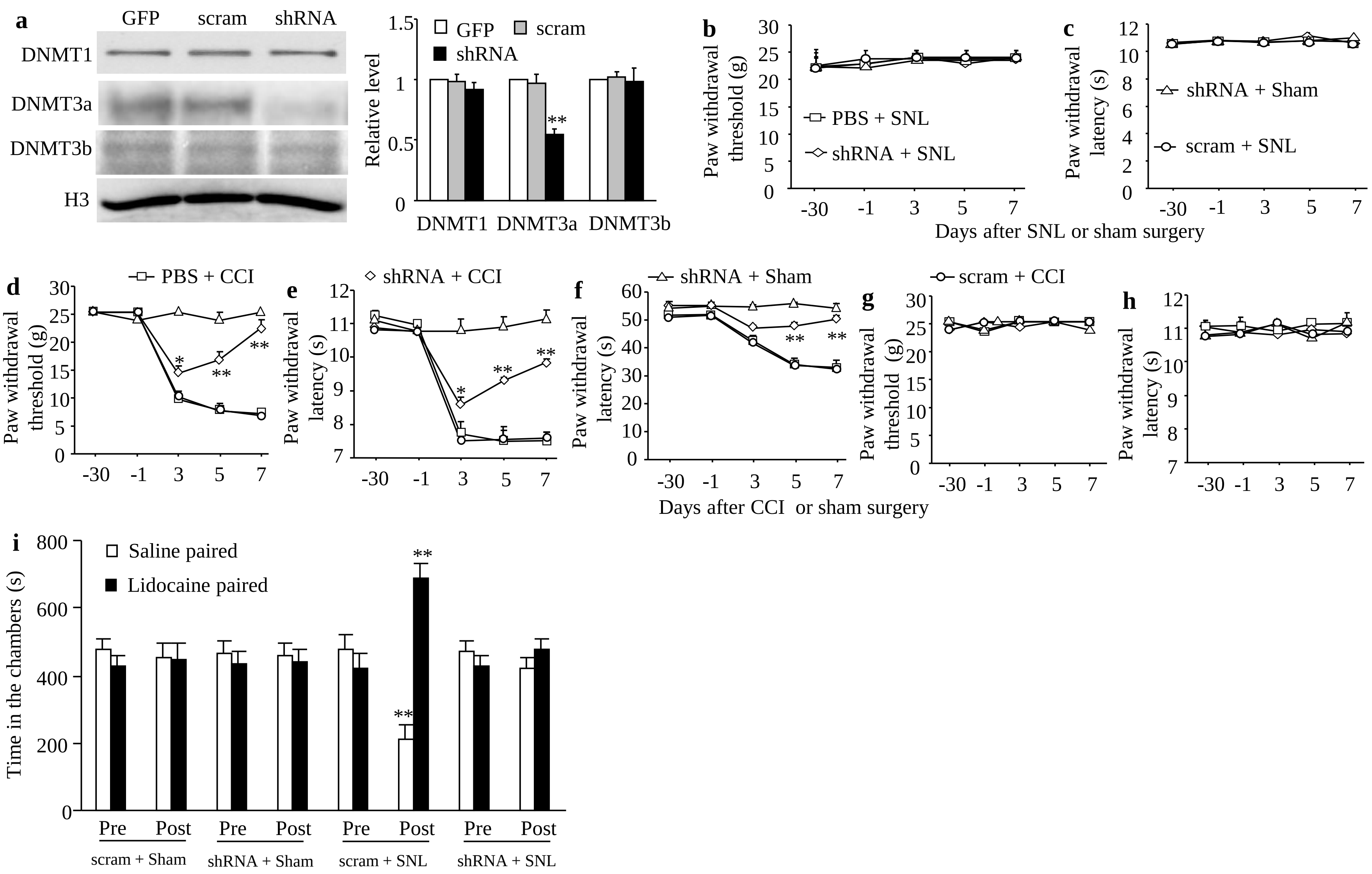

a
GFP
scram
shRNA
DNMT1
DNMT3a
DNMT3b
H3
Relative level
GFP
scram
shRNA
**
DNMT1
DNMT3a
DNMT3b
b
Paw withdrawal threshold (g)
PBS + SNL
shRNA + SNL
c
Paw withdrawal latency (s)
shRNA + Sham
scram + SNL
Days after SNL or sham surgery
d
PBS + CCI
shRNA + CCI
shRNA + Sham
scram + CCI
Paw withdrawal threshold (g)
e
Paw withdrawal latency (s)
f
Paw withdrawal latency (s)
g
Paw withdrawal threshold (g)
h
Paw withdrawal latency (s)
Days after CCI or sham surgery
i
Saline paired
Lidocaine paired
Time in the chambers (s)
Pre
Post
scram + Sham
shRNA + Sham
scram + SNL
shRNA + SNL

Supplementary Figure 2. (a) Levels of DNMT1, DNMT3a, and DNMT3b proteins in HEK-293T cells transfected with the vectors expressing *GFP* (control), scrambled *Dnmt3a* shRNA (scram), or *Dnmt3a* shRNA. n = 3 repeats/group. One-way ANOVA (relative level *vs*. group) followed by *post hoc* Tukey test, F_{group} (2, 8) = 0.88 for DNMT1, F_{group} (2, 8) = 23.2 for DNMT3a, and F_{group} (2, 8) = 0.13 for DNMT3b. ***P* < 0.01 *vs* the corresponding GFP-treated group. Full-length blots are presented in Supplementary Figure 6. (b, c) The effect of microinjection of AAV5-*Dnmt3a* shRNA (shRNA), AAV5-scrambled *Dnmt3a* shRNA (scram), or PBS into the ipsilateral L5 DRG on paw withdrawal responses to mechanical (b) and thermal (c) stimuli on the contralateral side at days shown before or after SNL or sham surgery in rats. n = 5 rats/group. Two-way ANOVA (effect *vs*. group × time interaction) followed by *post hoc* Tukey test, F_{group} (3, 124) = 0.13 for (b) and F_{group} (3, 124) = 0.63 for (c). (d-h) The effect of microinjection of AAV5-*Dnmt3a* shRNA (shRNA), AAV5-scrambled *Dnmt3a* shRNA (scram), or PBS into the unilateral L4 and L5 DRG on paw withdrawal responses to mechanical (d, g), thermal (e, h) and cold (f) stimuli on the ipsilateral (d, e, f) and contralateral (g, h) sides at days shown before or after CCI or sham surgery in rats. n = 5 rats/group. Two-way ANOVA (effect *vs*. group × time interaction) followed by *post hoc* Tukey test, F_{group} (3, 129) = 126.3 for (d), F_{group} (3, 129) = 79.4 for (e), F_{group} (3, 119) = 73.6 for (f), F_{group} (3, 129) = 0.39 for (g), and F_{group} (3, 129) = 1.33 for (h). **P* < 0.05 or ***P* < 0.01 *vs* the corresponding PBS plus CCI group. (i) The effect of microinjection of AAV5-*Dnmt3a* shRNA (shRNA) or AAV5-scrambled *Dnmt3a* shRNA (scram) into the ipsilateral L5 DRG on the duration of time spent in saline- or lidocaine-paired chambers on day 7 post-SNL or sham surgery in rats. Pre: preconditioning. Post: post-conditioning. n = 5 rats/group. ***P* < 0.01 *vs* the corresponding preconditioning by two-tailed paired Student's *t* test.

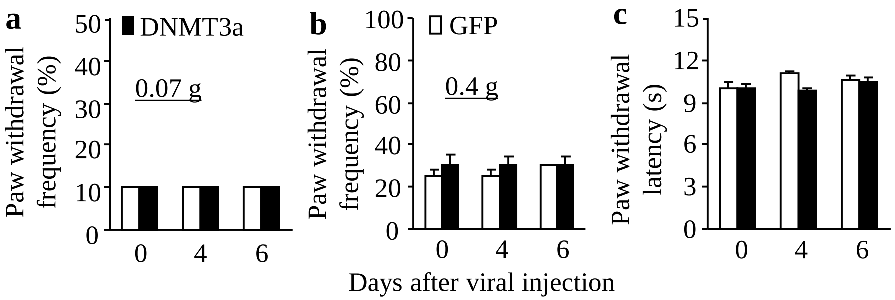

Supplementary Figure 3. Paw withdrawal responses to mechanical (a, b) and thermal (c) stimuli on the contralateral side at days as shown from control HSV-*GFP* (GFP)- or HSV-*Dnmt3a* (DNMT3a)-injected mice. n = 10/group. Two-way ANOVA (effect *vs.* group × time point interaction) followed by *post hoc* Tukey test, F_{group} (1, 23) = 0.00 for (a), F_{group} (1, 23) = 1.2 for (b), and F_{group} (1, 23) = 3 for (c).

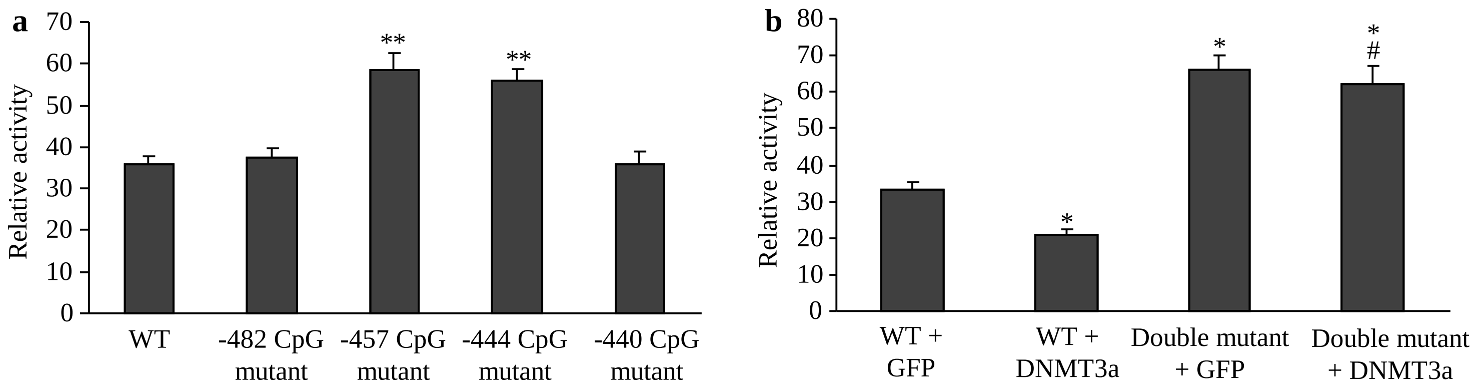

Supplementary Figure 4. (a) *Kcna2* gene promoter activity in HEK-293 cells transfected with wild type (WT) vector expressing the fragment (-622 to +74) of *Kcna2* gene or with the vectors expressing this fragment with a mutated base at the -482, -457, -444, or -440 CpG site. n = 3 repeats/treatment. One-way ANOVA (relative activity *vs.* group) followed by *post hoc* Tukey test, F_{group} (4, 14) = 137.9. ***P* < 0.01 *vs* the WT group. (b) *Kcna2* gene promoter activity in HEK-293 cells co-transfected with WT vector plus vector expressed GFP (GFP), WT vector plus vector expressing full-length *Dnmt3a* (DNMT3a), the vector expressing two mutated bases at the -457 and -444 CpG sites (double mutant) plus GFP, or the double mutant plus DNMT3a. n = 3 repeats/treatment. One-way ANOVA (relative activity *vs.* group) followed by *post hoc* Tukey test, F_{group} (3, 11) = 391.8. **P* < 0.05 *vs* the WT plus GFP group. #*P* < 0.05 *vs* the WT plus DNMT3a group.

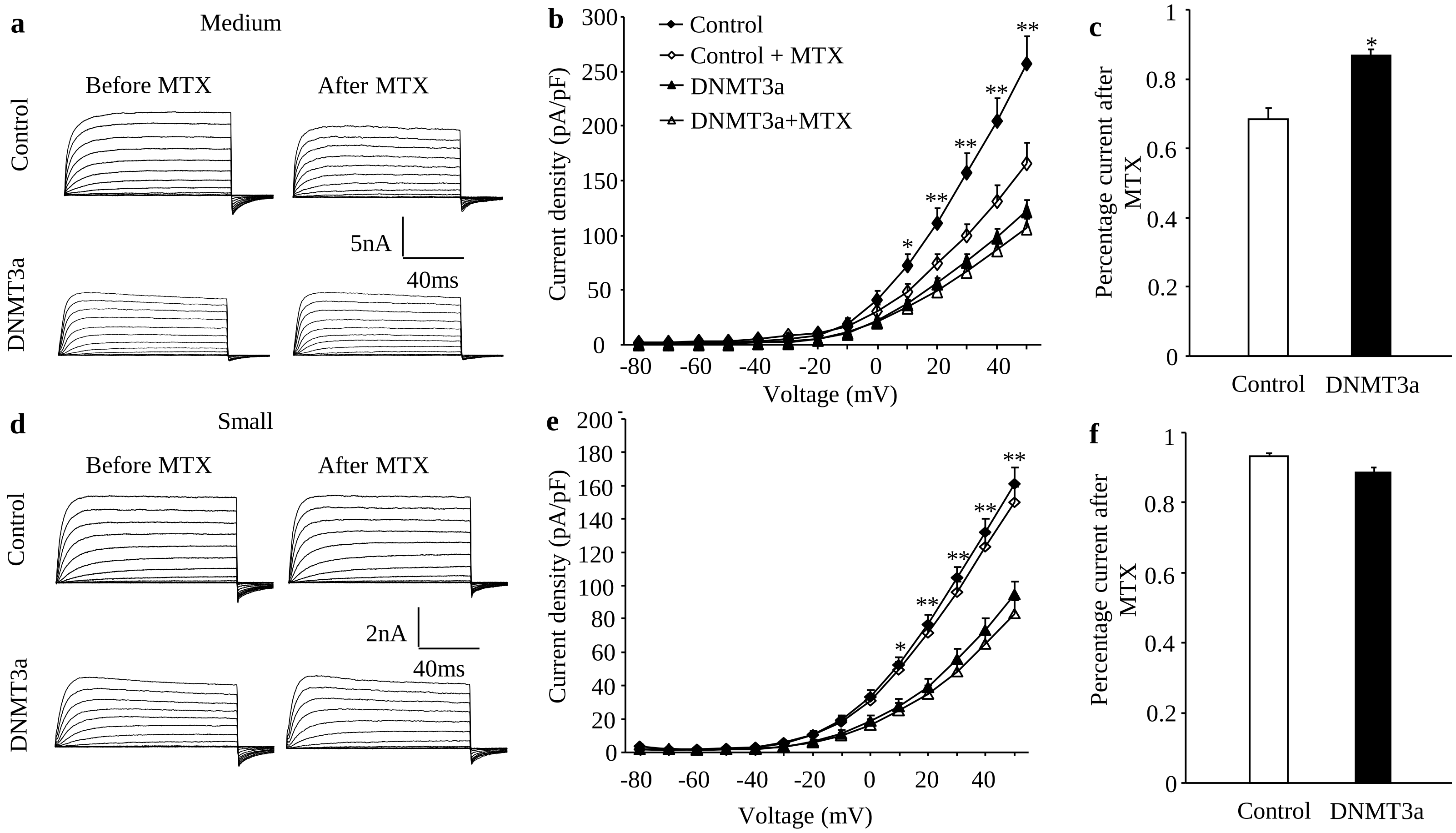
a
Medium
Before MTX
After MTX
Control
DNMT3a
5nA
40ms
b
Control
Control + MTX
DNMT3a
DNMT3a+MTX
Current density (pA/pF)
Voltage (mV)
c
Percentage current after MTX
Control
DNMT3a
d
Small
Before MTX
After MTX
Control
DNMT3a
2nA
40ms
e
Current density (pA/pF)
Voltage (mV)
f
Percentage current after MTX
Control
DNMT3a

Supplementary Figure 5. *Dnmt3a* overexpression reduces total Kv current density in medium and small neurons from the injected DRG 6–8 weeks after viral microinjection into the unilateral L4 and L5 DRG in rats. (a) Representative traces of total Kv current in medium DRG neurons from AAV5-*GFP* (Control)-treated and AAV5-*Dnmt3a* (DNMT3a)-treated groups before or after bath perfusion of 100 nM maurotoxin (MTX). (b) I-V curve for control (n = 22 neurons, 6 rats) and DNMT3a-treated (n = 27 neurons, 8 rats) medium DRG neurons before or after 100 nM MTX treatment. The current density was plotted against each voltage. One-way ANOVA (current density *vs.* group) followed by *post hoc* Tukey test, F_{group} (1, 13) = 50.8. *$P < 0.05$, **$P < 0.01$ *vs* the corresponding DNMT3a-treated group at the same voltage. (c) At +50 mV, the reduction in total Kv current after MTX treatment in medium DRG neurons was greater in the control group than in the DNMT3a-treated group. **$P < 0.01$ *vs* control group by two-tailed unpaired Student's *t*-test. (d) Representative traces of total Kv current in small DRG neurons from control and DNMT3a-treated groups before or after bath perfusion of 100 nM MTX. (e) I-V curve for control (n = 23 neurons, 8 rats) and DNMT3a-treated (n = 24 neurons, 9 rats) small DRG neurons before or after 100 nM MTX treatment. The current density was plotted against each voltage. One-way ANOVA (current density *vs.* group) followed by *post hoc* Tukey test, F_{group} (1, 13) = 68.7. *$P < 0.05$, **$P < 0.01$ *vs* the corresponding DNMT3a-treated group at the same voltage. (f) At +50 mV, the reduction in total Kv current after MTX treatment in small DRG neurons was similar between the control group and the DNMT3a-treated group.

Figure 2a

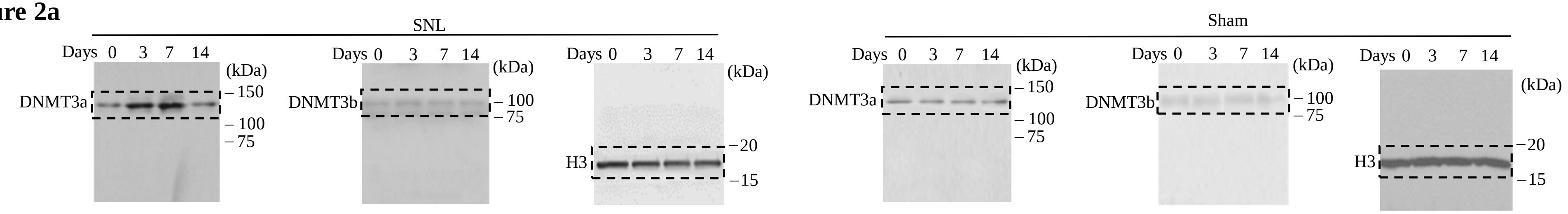

Figure 2e

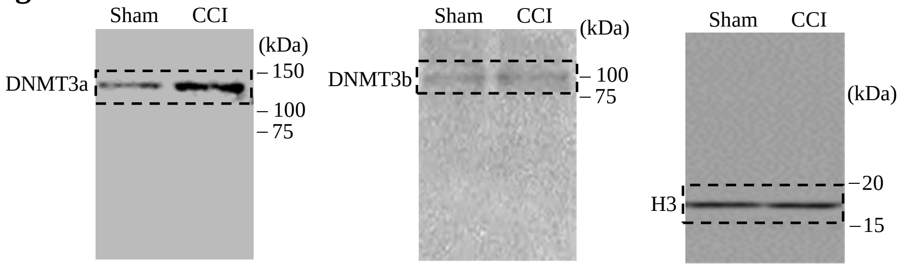

Figure 3b

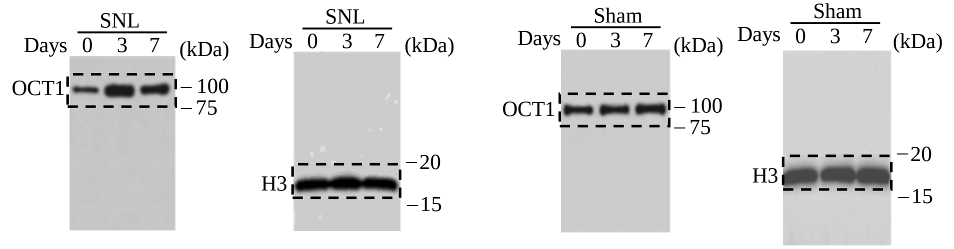

Figure 3d

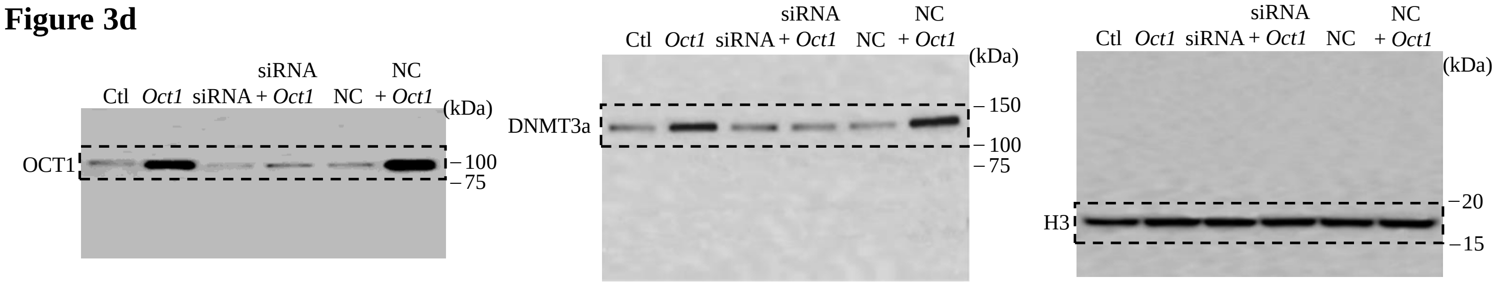

Figure 4b

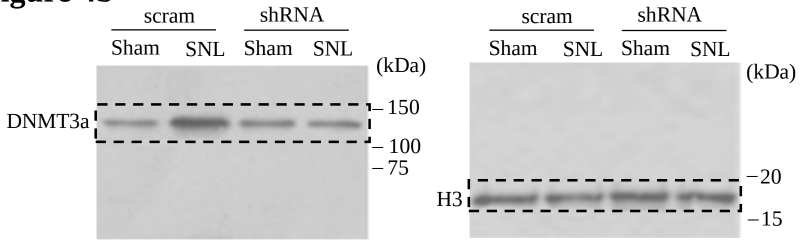

Figure 4b

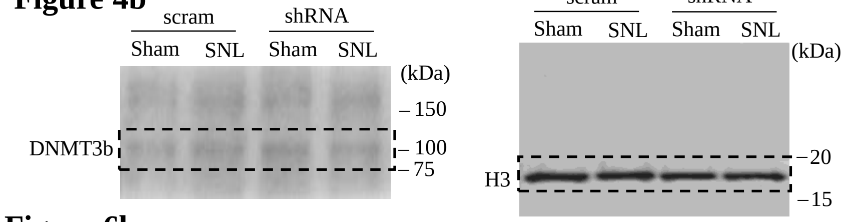

Figure 5b

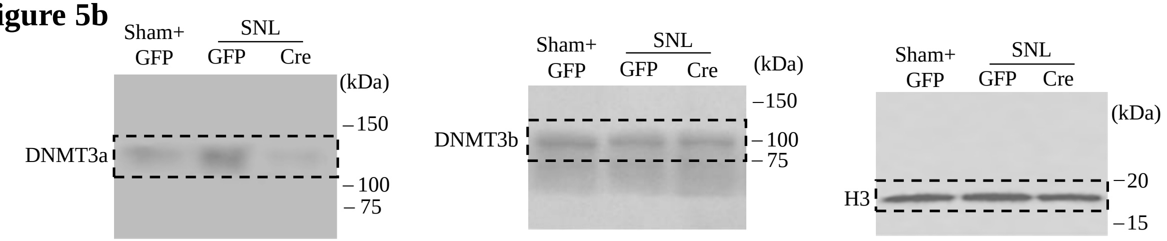

Figure 6b

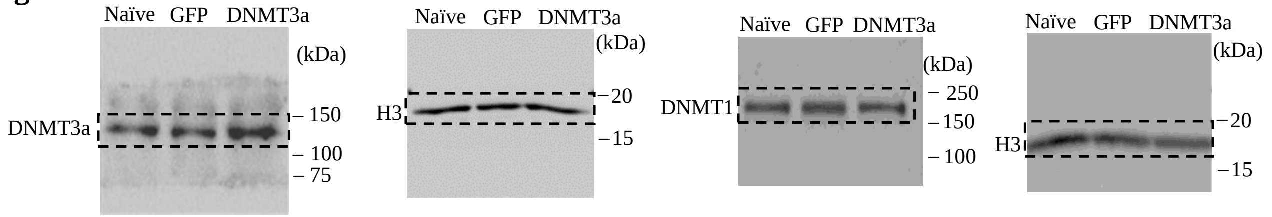

Figure 6j

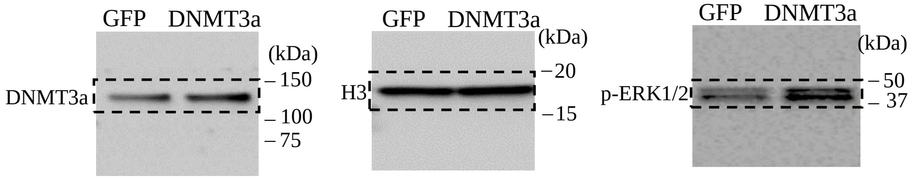

Figure 6j

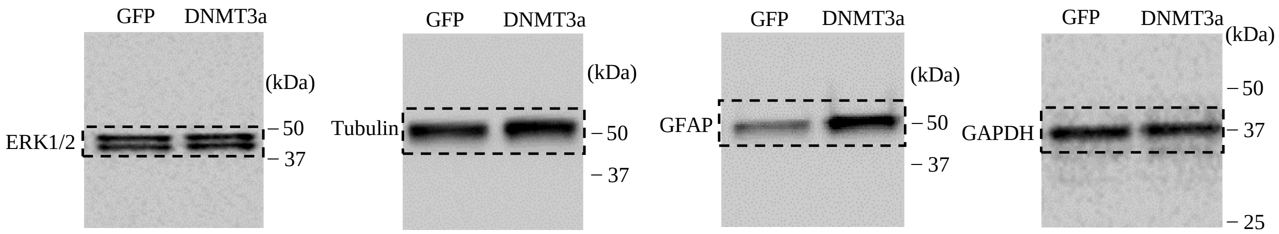

Figure 7b

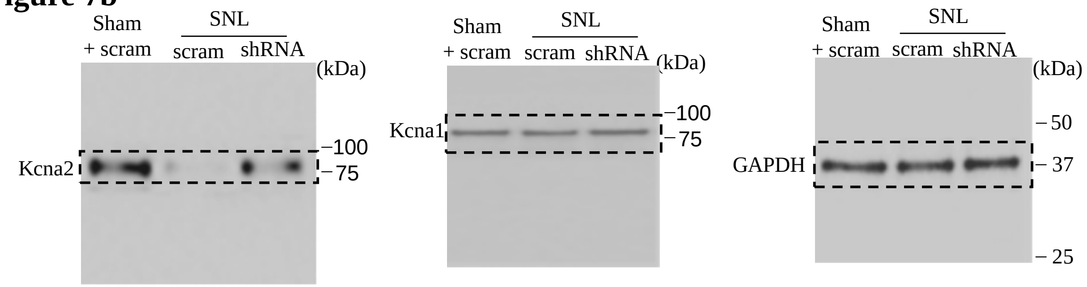

Figure 7d

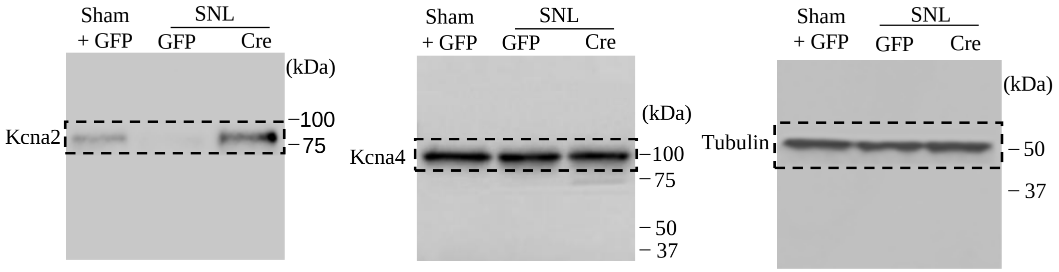

Figure 8b

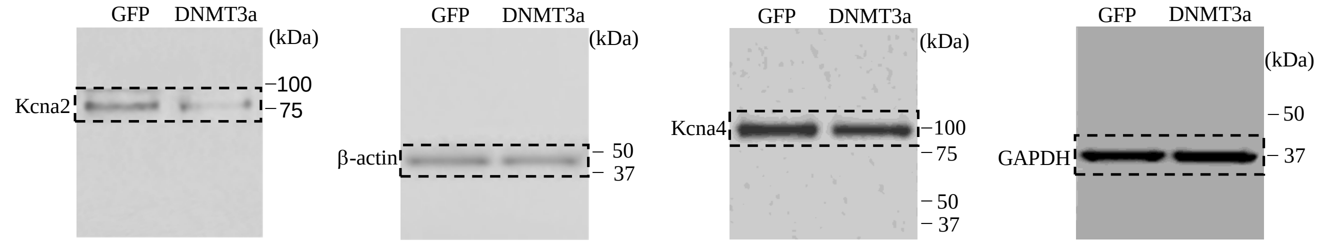

Supplementary Figure 1a

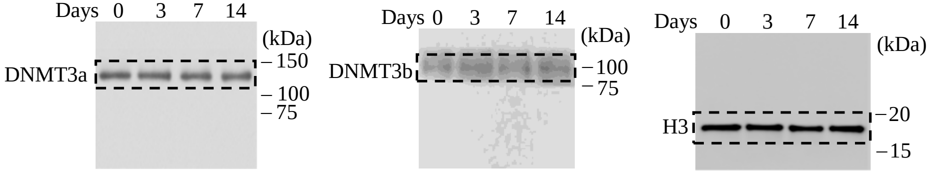

Supplementary Figure 1b

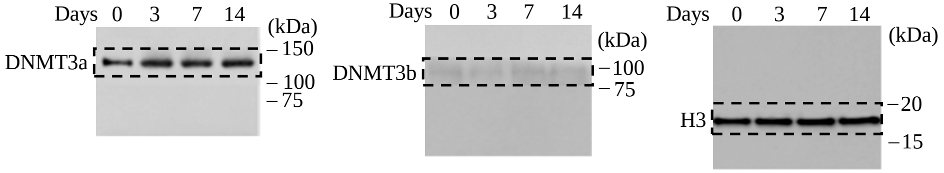

Supplementary Figure 1c

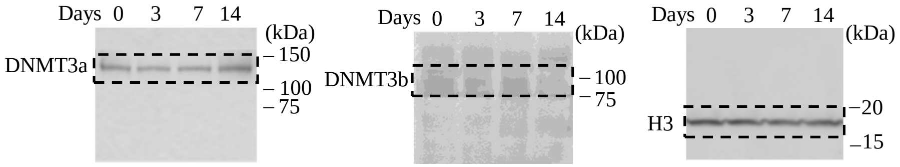

Supplementary Figure 1d

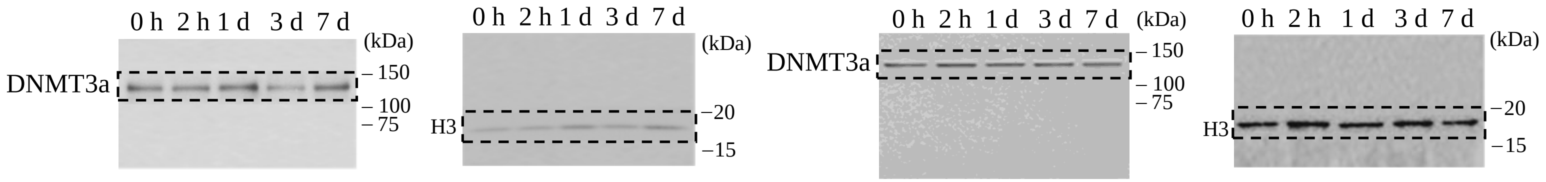

Supplementary Figure 1e

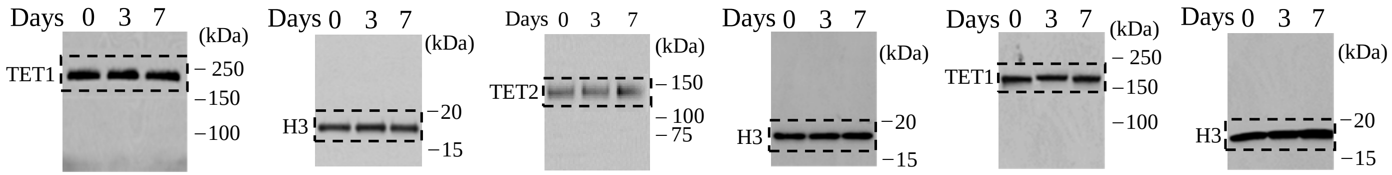

Supplementary Figure 1f

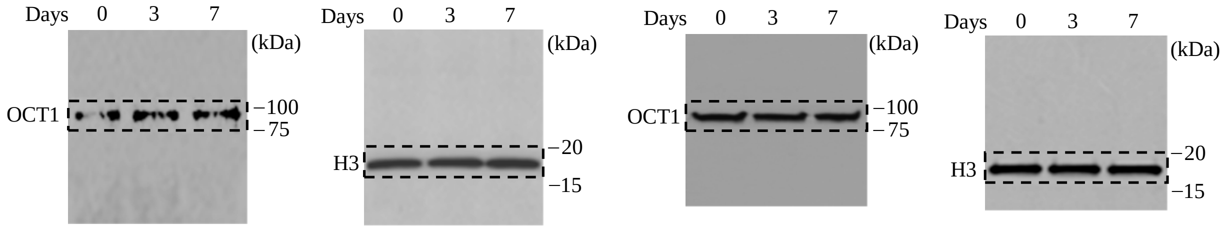

Supplementary Figure 2a

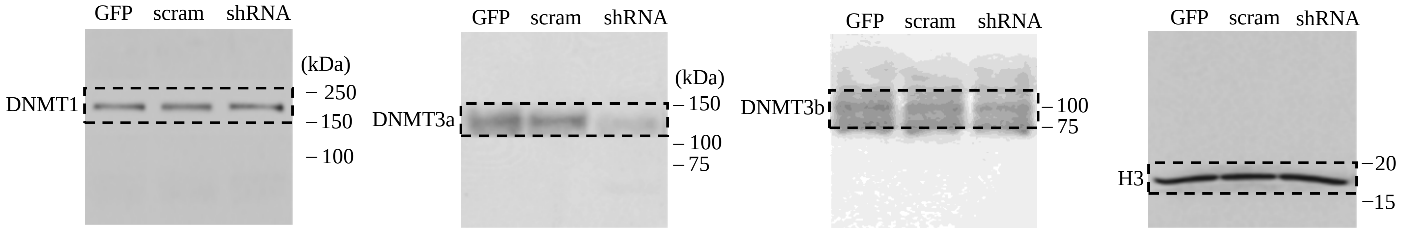

Supplementary Figure 6. Full-length pictures of the blots presented in the main figures and supplemental figures.

Supplementary Table 1. Locomotor test after viral injection

Treated groups	Placing	Grasping	Righting
PBS + SNL (rats)	5 (0)	5 (0)	5 (0)
AAV5-shRNA + SNL	5 (0)	5 (0)	5 (0)
AAV5-scram + SNL	5 (0)	5 (0)	5 (0)
AAV5-shRNA + Sham	5 (0)	5 (0)	5 (0)
AAV5-scram + Sham	5 (0)	5 (0)	5 (0)
PBS + CCI (rats)	5 (0)	5 (0)	5 (0)
AAV5-scram + CCI	5 (0)	5 (0)	5 (0)
AAV5-shRNA + CCI	5 (0)	5 (0)	5 (0)
PBS + SNL (mice)	5 (0)	5 (0)	5 (0)
AAV5-GFP + Sham	5 (0)	5 (0)	5 (0)
AAV5-GFP + SNL	5 (0)	5 (0)	5 (0)
AAV5-Cre + SNL	5 (0)	5 (0)	5 (0)
AAV5-Cre + Sham	5 (0)	5 (0)	5 (0)
AAV5-*Dnmt3a*	5 (0)	5 (0)	5 (0)
AAV5-GFP	5 (0)	5 (0)	5 (0)
HSV-*Dnmt3a*	5 (0)	5 (0)	5 (0)
HSV-GFP	5 (0)	5 (0)	5 (0)

N = 5-8/group; 5 trials; Mean (SEM)

Supplementary Table 2: Membrane input resistance and other action potential parameters in DRG neurons.

	Large Cell			Medium Cell			Small Cell		
	GFP	DNMT3a	t/p value	GFP	DNMT3a	t/p value	GFP	DNMT3a	t/p value
n	25cells, 5 rats	22cells, 5rats		25cells, 5rats	24cells, 5rats		13cells, 5rats	15cells, 5rats	
Rin, MΩ	45.40±2.62	50.45±3.36	-1.200/0.236	40.76±2.61	43.43±3.32	-0.634/0.529	47.17±4.51	53.99±8.11	-0.734/0.469
APT, mV	-13.68±0.96	-11.16±0.81	1.973/0.055	-14.40±0.94	-14.43±1.18	0.019/0.985	-16.43±1.71	-16.66±2.16	0.082/0.936
APO, mV	46.10±3.79	50.28±3.12	-0.838/0.407	48.17±3.02	48.52±3.15	-0.079/0.938	39.13±2.19	34.90±3.93	0.940/0.355
APA, mV	100.35±3.42	97.53±2.93	0.618/0.540	103.66±3.34	97.52±3.52	1.268/0.211	86.65±4.04	82.14±5.00	0.700/0.490
AHPA, mV	-19.19±1.14	-20.40±1.52	-0.650/0.519	-18.79±1.25	-18.49±1.31	-0.165/0.869	-14.86±0.86	-17.52±1.12	1.530/0.139

Values are Mean ± S.E.M., R_{in}: membrane input resistance. APT: action potential threshold. APA: action potential amplitude. APO: action potential overshoot. AHPA: afterhyperpolarization amplitude. All values are mean ± S.E.M. student t-test.

Supplementary Table 3 All primers used

**Names**	**Sequences (5'-3')**	**Names**	**Sequences (5'-3')**	**Names**	**Sequences (5'-3')**
PCR/Real-time PCR		***ChIP-PCR***		rKCNA2-N-F3	GGTTAGGGTATTTATTTGTAAGGGTAA
RT-rat KCNA2	GGGTGACTCTCATCTTTGGA	rKCNA2-ChIP-F1	GGGGCAAAGACAACAGTGAAT	rKCNA2-N-R3	CTATACAATCAACTACACTTCCCATCA
RT-mouse KCNA2	GTCCCCGTCACATCTTCTCAC	rKCNA2-ChIP-R1	CCAACATAATGAAGTAGGCAGG	rKCNA2-F4	TTTGTGGTTTGTGYGTGATATA
Rat-KCNA2-F	CCCATCTGCAAGGGCAACGT	rKCNA2-ChIP-F2	CTGTCTGGCAGCATCGTAATAA	rKCNA2-R4	CCTAAACTCTAAAAATACAAAACAAT
Rat-KCNA2-R	CACAGCCTCCTTTGGCTGGC	rKCNA2-ChIP-R2	CACTGCCACCACTGAGCTGT	rKCNA2-N-F4	GTGATGGGAAGTGTAGTTGAT
Rat-DNMT3A-F	GTGGTTCGGAGATGGCAAAT	rKCNA2-ChIP-F3	GGGGATCTAACCCAATGGAC	rKCNA2-N-R4	ACCCCAAATCTAACTTCCTCTTC
Rat-DNMT3A-R	TGGAGGACTTCGTAGATGGCT	rKCNA2-ChIP-R3	TGGGTGATAGACAGGGCAGG		
Rat-DNMT3B-F	CGCAGGACGTGGTAGGAGATG	rKCNA2-ChIP-F4	CTGCCTGCCCTGTCTATCAC	***Cloning***	
Rat-DNMT3B-R	ACTGTTGCTGTTTCGGGTTC	rKCNA2-ChIP-R4	CCCATTCCATGCACTCTTCT	rOCT1-F	AAATGGCGGACGGAGGAG
Rat-DNMT1-F	CGAGTGCCTGCACTTGCC	rKCNA2-ChIP-F5	ACCCCTGATGTCGGCCTAGA	rOCT1-R	ACAGGCGTCATGTTTTAGCC
Rat-DNMT1-R	TCCTCTTTATGTAGTTTGGTTTCC	rKCNA2-ChIP-R5	GCAGAGAAGCCCATTTTACT	rOCT1-N-F	ATATCTAGAGCCACCATGGCGGACGGAGG
Rat-OCT1-F	CACTTCCACAGAGCCAGACA	rDNMT3A-ChIP-F	ACCCAAAGAGTGCAGCAAAC	rOCT1-N-R	CGATAGGATCCTCACTGTGCCTTGGAGGC
Rat-OCT1-R	GGTGGTTTGGCTGAAGTCAT	rDNMT3A-ChIP-R	GTGGAGGAGGAAGTGAGCAG	rDNMT3A-Luci-F	TATAGGTACCAGGAACCGTTGTGTGTCTCC
Rat-GAPDH-F	TCGGTGTGAACGGATTTGGC			rDNMT3A-Luci-R	TCGCAAGCTTTCGTGGTCTTTGTGAGCAAG
Rat-GAPDH-R	CCTTCAGGTGAGCCCCAGC	***Bisulfite Sequencing***		rDNMT3AshRNA-F	TTACCGGTGGGCAGGAAGAGGGCCTATTTC<br>CCATGA
Mouse-KCNA2-F	CTGCAAGGGCAACGTCACAC	rKCNA2-F1	GTAGAAATAGAAGATTGGAGTGG		
Mouse-KCNA2-R	GGGACAGTGAGATGCTTGGC	rKCNA2-R1	CTAAAACCAAAACTAACRAAATA	rDNMT3A-shRNA-R	AATCTAGACATCCACTGTGAATGATAACGA<br>ATTCACTTATCATTCACAGTGGATGGGATCC<br>TCGTCCTTTCCACAAGATAT
Mouse-DNMT3A-F	GCCAAGAAACCCAGAAAGAGC	rKCNA2-N-F1	GGTTTGTAGATTTTGTTTGGTAGT		
Mouse-DNMT3A-R	GCACATGCCTCCAATGAAGA	rKCNA2-N-R1	CACTACCACCACTAAACTATCCAT	rKCNA2-Luci-F	CGACGCGTCGCCTTCACCTGCTGTATTGCCC<br>TGGGAA
Mouse-DNMT3B-F	CAGCCTTCTGAATTACACGCA	rKCNA2-F2	TAGGGTTTTATTAAAGAATGGGGT		
Mouse-DNMT3B-R	TCCCATTGCTATGTCGGGTT	rKCNA2-R2	ACACTAATTTACCCTTACAAAT	rKCNA2-Luci-R	GAAGATCTTCTTGCAGATGGGTACCCGAGC<br>CTCCAT
Mouse-DNMT1-F	AGTCGGACAGTGACACCCTTT	rKCNA2-N-F2	ATAAGGGGATTTAATTTAATGGAT		
Mouse-DNMT1-R	TGTGTCTACAACTCTGCGTTTCT	rKCNA2-N-R2	TACCCCAACCTCCATATTCCTAAA		
Mouse-GAPDH-F	TCGGTGTGAACGGATTTGGC	rKCNA2-F3	GTTTATTATTTAGGAATATGGAGG		
Mouse-GAPDH-R	TCCCATTCTCGGCCTTGACT	rKCNA2-R3	AATCCTACCCACCCACTAT		

RT: Reverse-transcription. F: Forward. R: Reverse. N-PCR: Nested PCR. Underlined letters: the restriction enzyme recognition sites.
